# Supplementary material for: Exploring burnout, perfectionism, and moral injury among UK physiotherapists: A qualitative study on professional fulfilment and well-being
Source: PLoS One. 2025 Feb 13;20(2):e0313730. doi: 10.1371/journal.pone.0313730 (PMC11825001; doi:10.1371/journal.pone.0313730)
Supplement: S1 File — (PDF) [file pone.0313730.s001.pdf]

Supplementary Document on codes, Categories and Overarching themes formation

Theme 1: Physiotherapy Under Pressure: Workload, Burnout, and Perfectionism

Burnout and Exhaustion

- Emotional Exhaustion
- Low Energy and Performance
- Feeling Overwhelmed
- Physical Exhaustion

The aftermath of COVID-19

- COVID-19 Impact on Mental Health
- Increased Workload After COVID-19
- Anxiety After COVID-19
- Impact of Pandemic on Mental Health
- Transition to Remote Work

Work Environment

- Inconsistent Management Support
- Bureaucratic Challenges
- Need for Organizational Change
- Career Stages

Perfectionism

- Burnout and Perfectionism
- Seeking Professional Help
- Coping with Perfectionism

Moral Injury

- Conflict Between Values and Reality
- Impact on Professional Integrity
- Emotional and Psychological Consequences

Theme 2: Interpersonal Dynamics and Support Systems

Team Dynamics

- Team Support and Dynamics
- Managers' Understanding
- Team Interaction
- Collaboration and Communication

Support Networks

- Support from Colleagues
- Emotional Support Systems
- Support from Family and Friends
- Peer Support

Interpersonal Challenges

- Being Criticized
- Being Bullied
- Minimal Team Interaction
- Conflict with Colleagues

Emotional Impact

- Emotional Support
- Impact on Personal Relationships
- Feelings of Isolation
- Emotional Dysregulation

Theme 3: Professional Fulfilment and Identity

Recognition and Appreciation

- Recognition by Patients and Colleagues
- Appreciation in Work
- Importance of Job Recognition
- Positive Outcomes

Sense of Accomplishment

- Feeling of Personal Productivity and Usefulness
- Confidence in Professional Role
- Professional Satisfaction

Career Development and Growth

- Need for Continuous Professional Development
- Working Outside Role to Create Opportunities
- No Career Progression Opportunities

Career Stagnation and Change

- Desire for Career Change
- Being in the Same Role for a While

Theme 4: Work-Life Balance and Well-being

Work-Life Integration and Flexibility

- Poor Work-Life Balance
- Struggle with Maintaining Work-Life Balance
- Work-Life Integration
- Impact of Work on Personal Life
- Setting Boundaries
- Flexibility in Scheduling
- Taking Breaks
- Support and Resources

Personal Well-being and Coping Mechanisms

- Mental Health
- Physical Health Symptoms
- Sleep Issues
- Emotional Resilience
- Flexibility and Boundaries
- Exercise as Coping Mechanism
- Therapy and Self-Awareness
- Seeking Professional Help
- Strategies for Stress Management

Management Respecting Work-Life Balance

- Support from Family and Friends
- Need for Better Mental Health Support
- Resilience Building
