# Supplementary material for: Exploring burnout, perfectionism, and moral injury among UK physiotherapists: A qualitative study on professional fulfilment and well-being
Source: PLoS One. 2025 Feb 13;20(2):e0313730. doi: 10.1371/journal.pone.0313730 (PMC11825001; doi:10.1371/journal.pone.0313730)
Supplement: S2 File — (PDF) [file pone.0313730.s002.pdf]

COREQ Checklist

Exploring Burnout, Perfectionism, and Moral Injury Among UK Physiotherapists: A Qualitative Study on Professional Fulfilment and Well-being

Domain	Criterion	Response with Page and Line Numbers
Domain 1: Research Team and Reflexivity	1. Interviewer/facilitator identified?	Lead author conducted interviews (p. 5, lines 12-14).
	2. Credentials of interviewer	Researcher’s PhD credentials listed (p. 2, lines 4-6).
	3. Occupation of interviewer	Researcher’s role as qualitative researcher stated (p. 2, line 5).
	4. Gender of interviewer	Gender provided (p. 5, line 16).
	5. Experience and training	Experience in qualitative research stated (p. 5, lines 12-13).
	6. Relationship established	Relationship built prior to interviews (p. 5, lines 18-20).
	7. Participants' knowledge of interviewer	Participants informed of researcher’s goals and background (p. 5, lines 21-23).
	8. Interviewer characteristics	Biases noted in reflective journal (p. 6, lines 1-4).
Domain 2: Study Design	9. Methodological orientation	Framework analysis employed (p. 6, lines 12-14).
	10. Sampling strategy	Purposive sampling used, focusing on burnout experiences (p. 6, lines 16-18).
	11. Method of approach	Participants invited via email (p. 6, line 20).
	12. Sample size	Twelve participants included (p. 6, line 22).
	13. Non-participation	No dropouts reported (p. 7, line 2).
	14. Setting of data collection	Interviews conducted via Zoom (p. 7, lines 4-6).
	15. Presence of non-participants	No non-participants were present (p. 7, line 8).
	16. Description of sample	Participant demographics detailed (p. 7, lines 10-15).
	17. Interview guide	Semi-structured guide developed through pilot study (p. 7, lines 18-21).
	18. Repeat interviews	No repeat interviews; saturation achieved (p. 8, lines 2-3).
	19. Audio/visual recording	Interviews were audio-recorded (p. 8, line 5).
	20. Field notes	Field notes taken during and after interviews (p. 8, lines 7-9).
	21. Duration of interviews	Each interview lasted 45-60 minutes (p. 8, line 11).
	22. Data saturation	Data saturation confirmed (p. 8, lines 12-14).

	23. Transcripts returned	Transcripts not returned to participants (p. 8, line 16).
Domain 3: Analysis and Findings	24. Number of data coders	One primary coder with peer review (p. 9, lines 3-5).
	25. Description of coding tree	Coding tree detailed in NVivo (p. 9, lines 7-9).
	26. Derivation of themes	Themes derived inductively from data (p. 9, lines 10-12).
	27. Software	NVivo 14 used for data management (p. 9, line 14).
	28. Participant checking	No participant feedback on findings (p. 9, lines 15-17).
	29. Quotations presented	Participant quotations included to illustrate themes (p. 10, lines 2-4).
	30. Data and findings consistency	Data presented consistently with findings (p. 10, lines 5-8).
	31. Clarity of major themes	Major themes outlined clearly in findings section (p. 10, lines 10-12).
	32. Clarity of minor themes	Minor themes discussed, diverse cases highlighted (p. 10, lines 13-16).
