# Supplementary material for: Exploring burnout, perfectionism, and moral injury among UK physiotherapists: A qualitative study on professional fulfilment and well-being
Source: PLoS One. 2025 Feb 13;20(2):e0313730. doi: 10.1371/journal.pone.0313730 (PMC11825001; doi:10.1371/journal.pone.0313730)
Supplement: Attachment — Submitted filename: PONE-D-24-48686.docx [file pone.0313730.s003.docx]

PLOS ONE

# Exploring Burnout, Perfectionism, and Moral Injury Among UK Physiotherapists: A Qualitative Study on Professional Fulfilment and Well-being

--Manuscript Draft--

| Manuscript Number: | PONE-D-24-48686 |
| --- | --- |
| Article Type: | Research Article |
| Full Title: | Exploring Burnout, Perfectionism, and Moral Injury Among UK Physiotherapists: A Qualitative Study on Professional Fulfilment and Well-being |
| Short Title: | Burnout Qualitative Study |
| Corresponding Author: | Sivaramkumar Shanmugam, Ph.D Glasgow Caledonian University  UNITED KINGDOM OF GREAT BRITAIN AND NORTHERN IRELAND |
| Keywords: | burnout; Perfectionism; Moral Injury; Physiotherapists; Work-Life Balance; Professional Fulfilment; Support Systems; qualitative study |
| Abstract: | Background  Burnout, perfectionism, and moral injury are prevalent issues among healthcare professionals, including physiotherapists. The demanding nature of the profession, compounded by high workloads, emotional and physical exhaustion, and systemic challenges, has significant implications for the well-being and professional satisfaction of physiotherapists. This study aimed to explore these issues, by providing a qualitative exploration of UK physiotherapists' lived experiences.  Objectives  To explore the lived experiences of UK physiotherapists regarding burnout, perfectionism, and moral injury, and to develop a comprehensive understanding of the personal and professional impacts of these issues to inform the development of effective support systems and interventions.  Methods  This qualitative exploratory study involved semi-structured interviews with 12 UK physiotherapists. Framework approach was used to identify key themes and patterns in the data, providing a nuanced understanding of the challenges faced by physiotherapists.  Results  Four primary themes emerged: (1) Physiotherapy Under Pressure: Workload, Burnout, and Perfectionism, (2) Interpersonal Dynamics and Support Systems, (3) Professional Fulfilment and Identity, and (4) Work-Life Balance and Well-being. Physiotherapists reported high levels of burnout and exhaustion due to relentless workloads, exacerbated by and after the COVID-19 pandemic. Perfectionism further contributed to emotional exhaustion and feelings of inadequacy. Inconsistent management support, bureaucratic challenges, and a lack of career progression opportunities were significant stressors. Effective team dynamics and support systems were crucial in mitigating stress, yet many faced interpersonal challenges such as criticism and bullying. Achieving work-life balance was a persistent struggle, highlighting the need for organisational changes to support flexibility and well-being.  Conclusion  Burnout, perfectionism, and moral injury significantly impact physiotherapists' well- being and professional satisfaction. Addressing these issues requires systemic changes within healthcare organisations to provide robust support systems, flexible working conditions, and opportunities for professional development. |

| Order of Authors: | Glykeria Skamagki, PhD |
| --- | --- |
|  | Laura Blackburn, DPT |
|  | Daniel Biggs, DPT |
|  | Maria Kolitsida, MSc |
|  | Cameron Black, ProfD |
|  | Sivaramkumar Shanmugam, Ph.D |
| Additional Information: |  |
| Question | Response |
| Financial Disclosure  Enter a financial disclosure statement that describes the sources of funding for the work included in this submission. Review the [submission guidelines](http://journals.plos.org/plosone/s/submission-guidelines#loc-financial-disclosure-statement) for detailed requirements. View published research articles from [*PLOS ONE*](https://journals.plos.org/plosone/) for specific examples.  This statement is required for submission and will appear in the published article if the submission is accepted. Please make sure it is accurate. | The author(s) received no specific funding for this work. |
| Funded studies  Enter a statement with the following details:   - Initials of the authors who received each award - Grant numbers awarded to each author - The full name of each funder - URL of each funder website - Did the sponsors or funders play any role in the study design, data collection and analysis, decision to publish, or preparation of the manuscript? |  |
| Did you receive funding for this work? |  |
| Competing Interests  Use the instructions below to enter a competing interest statement for this submission. On behalf of all authors, disclose any [competing interests](http://journals.plos.org/plosone/s/competing-interests) that could be perceived to bias this work—acknowledging all financial support and any other relevant financial or non- financial competing interests. | The authors have declared that no competing interests exist. |

| This statement is required for submission and will appear in the published article if the submission is accepted. Please make sure it is accurate and that any funding sources listed in your Funding Information later in the submission form are also declared in your Financial Disclosure statement.  View published research articles from  [*PLOS ONE*](http://journals.plos.org/plosone/) for specific examples. |  |
| --- | --- |
| NO authors have competing interests  Enter: *The authors have declared that no competing interests exist.*  Authors with competing interests  Enter competing interest details beginning with this statement:  *I have read the journal's policy and the authors of this manuscript have the following competing interests: [insert competing interests here]* |  |
| * typeset |  |
| Ethics Statement  Enter an ethics statement for this submission. This statement is required if the study involved:   - Human participants - Human specimens or tissue - Vertebrate animals or cephalopods - Vertebrate embryos or tissues - Field research   Write "N/A" if the submission does not require an ethics statement.  General guidance is provided below. Consult the [submission guidelines](http://journals.plos.org/plosone/s/submission-guidelines#loc-guidelines-for-specific-study-types) for detailed instructions. Make sure that all | The School of Health and Life Sciences - Ethics Committee. Ethics approval number HLS/PSWAHS/22/017. Participants agreed and consented to take part at the end of the quantitative survey in the first study – ‘Physiotherapy Under Pressure: A Cross- Sectional Study on The Interplay Between Perfectionism, Moral Injury, and Burnout.’ At the end of the survey, participants were asked if they were interested in taking part in a subsequent qualitative study. If they expressed interest, they were prompted to provide their name and email address.  Recruitment and interviews for the qualitative study were carried out from the start of October 2023 to the end of January 2024. Participants were informed that consent was an ongoing process and that they could withdraw at any time. This information was reiterated when the participant information sheet was emailed during the recruitment process for the qualitative study. The explicit information included the purpose of the study, assurances of anonymity and confidentiality, and an emphasis on the voluntary nature of participation. Written consent was sought at this stage.  During the interview, consent was sought again at the outset. Participants provided verbal consent, and it was documented by the researcher at the interview. Following explicit consent, the interview proceeded. At the conclusion of the interview, participants were given information on how to reach out to the research team, along with suggestions for post-interview resources and mental health support. |

| information entered here is included in the Methods section of the manuscript. |  |
| --- | --- |
| Format for specific study types  Human Subject Research (involving human participants and/or tissue)   - Give the name of the institutional review board or ethics committee that approved the study - Include the approval number and/or a statement indicating approval of this research - Indicate the form of consent obtained (written/oral) or the reason that consent was not obtained (e.g. the data were analyzed anonymously)   Animal Research (involving vertebrate animals, embryos or tissues)   - Provide the name of the Institutional Animal Care and Use Committee (IACUC) or other relevant ethics board that reviewed the study protocol, and indicate whether they approved this research or granted a formal waiver of ethical approval - Include an approval number if one was obtained - If the study involved *non-human primates*, add *additional details* about animal welfare and steps taken to ameliorate suffering - If anesthesia, euthanasia, or any kind of animal sacrifice is part of the study, include briefly which substances and/or methods were applied   Field Research  Include the following details if this study involves the collection of plant, animal, or other materials from a natural setting:   - Field permit number - Name of the institution or relevant body that granted permission |  |
|  |  |
| Data Availability  Authors are required to make all data underlying the findings described fully available, without restriction, and from the time of publication. PLOS allows rare exceptions to address legal and ethical | Yes - all data are fully available without restriction |

| concerns. See the [PLOS Data Policy](http://journals.plos.org/plosone/s/data-availability) and [FAQ](http://journals.plos.org/plosone/s/data-availability#loc-faqs-for-data-policy) for detailed information.  A Data Availability Statement describing where the data can be found is required at submission. Your answers to this question constitute the Data Availability Statement and will be published in the article, if accepted. |  |
| --- | --- |
| Important: Stating ‘data available on request from the author’ is not sufficient. If your data are only available upon request, select ‘No’ for the first question and explain your exceptional situation in the text box. |  |
| Do the authors confirm that all data underlying the findings described in their manuscript are fully available without restriction? |  |
| Describe where the data may be found in full sentences. If you are copying our sample text, replace any instances of XXX with the appropriate details.   - If the data are held or will be held in a public repository, include URLs, accession numbers or DOIs. If this information will only be available after acceptance, indicate this by ticking the box below. For example: *All XXX files are available from the XXX database (accession number(s) XXX, XXX.*). - If the data are all contained within the manuscript and/or Supporting Information files, enter the following: *All relevant data are within the manuscript and its Supporting Information files.* - If neither of these applies but you are able to provide details of access elsewhere, with or without limitations, please do so. For example:   *Data cannot be shared publicly because of [XXX]. Data are available from the XXX Institutional Data Access / Ethics Committee (contact via XXX) for researchers who meet the criteria for access to confidential data.* | All relevant data are within the manuscript and its Supporting Information files. |

| *The data underlying the results presented in the study are available from (include the name of the third party and contact information or URL).*   - This text is appropriate if the data are owned by a third party and authors do not have permission to share the data.   * typeset |  |
| --- | --- |
| Additional data availability information: |  |

Manuscript	[Click here to access/download;Manuscript;EXPLOR~1.DOC](https://www2.cloud.editorialmanager.com/pone/download.aspx?id=38280707&guid=d81ad0ec-99c5-429a-8a3c-7f46a799cb2f&scheme=1)[<https://www2.cloud.editorialmanager.com/pone/download.aspx?id=38280707&guid=d81ad0ec-99c5-429a-8a3c-7f46a799cb2f&scheme=1>](https://www2.cloud.editorialmanager.com/pone/download.aspx?id=38280707&guid=d81ad0ec-99c5-429a-8a3c-7f46a799cb2f&scheme=1)

1 **Exploring Burnout, Perfectionism, and Moral Injury Among UK**
2 **Physiotherapists: A Qualitative Study on Professional Fulfilment and**
3 **Well-being**

4

5 Glykeria Skamagki^1^, Laura Blackburn^2^, Daniel Biggs^3^, Maria Kolitsida^1^, Cameron
6 Black^4^, Sivaramkumar Shanmugam^2^
7 ^1^ School of Sport, Exercise and Rehabilitation Sciences, College of Life and
8 Environmental Sciences, University of Birmingham, Birmingham, United Kingdom
9 ^2^ Department of Physiotherapy and Paramedicine, School of Health and Life Sciences,
10 Glasgow Caledonian University, Glasgow, United Kingdom
11 ^3^ Academy of Sport and Wellbeing, University of Highlands and Islands, UHI Perth,
12 Perth,
13 United Kingdom
14 ^4^ Division of Occupational Health, Buckinghamshire Healthcare NHS Trust, Aylesbury,
15 United
16 Kingdom

17

18 Corresponding Author
19 [Sivaram.shanmugam@gcu.ac.uk](mailto:Sivaram.shanmugam@gcu.ac.uk)

20

21

22

23

24

25

26

27 **Exploring Burnout, Perfectionism, and Moral Injury Among UK**
28 **Physiotherapists: A Qualitative Study on Professional Fulfilment and Well-being**
29 **Abstract**
30 Background: Burnout, perfectionism, and moral injury are prevalent issues among
31 healthcare professionals, including physiotherapists. The demanding nature of the
32 profession, compounded by high workloads, emotional and physical exhaustion, and
33 systemic challenges, has significant implications for the well-being and professional
34 satisfaction of physiotherapists. This study aimed to explore these issues, by providing
35 a qualitative exploration of UK physiotherapists' lived experiences.
36 Objectives: To explore the lived experiences of UK physiotherapists regarding burnout,
37 perfectionism, and moral injury, and to develop a comprehensive understanding of the
38 personal and professional impacts of these issues to inform the development of
39 effective support systems and interventions.
40 Methods: This qualitative exploratory study involved semi-structured interviews with 12
41 UK physiotherapists. Framework approach was used to identify key themes and
42 patterns in the data, providing a nuanced understanding of the challenges faced by
43 physiotherapists.
44 Results: Four primary themes emerged: (1) Physiotherapy Under Pressure: Workload,
45 Burnout, and Perfectionism, (2) Interpersonal Dynamics and Support Systems, (3)
46 Professional Fulfilment and Identity, and (4) Work-Life Balance and Well-being.
47 Physiotherapists reported high levels of burnout and exhaustion due to relentless
48 workloads, exacerbated by and after the COVID-19 pandemic. Perfectionism further
49 contributed to emotional exhaustion and feelings of inadequacy. Inconsistent
50 management support, bureaucratic challenges, and a lack of career progression
51 opportunities were significant stressors. Effective team dynamics and support systems
52 were crucial in mitigating stress, yet many faced interpersonal challenges such as
53 criticism and bullying. Achieving work-life balance was a persistent struggle,
54 highlighting the need for organisational changes to support flexibility and well-being.
55 **Conclusion:** Burnout, perfectionism, and moral injury significantly impact
56 physiotherapists' well-being and professional satisfaction. Addressing these issues
57 requires systemic changes within healthcare organisations to provide robust support
58 systems, flexible working conditions, and opportunities for professional development.
59 **Keywords:** Burnout, Perfectionism, Moral Injury, Physiotherapists, Work-Life Balance,
60 Professional Fulfilment, Support Systems, Qualitative Study

## 61 Introduction

62 Burnout is increasingly prevalent among physiotherapists and has been described as
63 a critical issue with significant implications for both healthcare professionals and the
64 quality of patient care (1). Burnout is characterised by emotional exhaustion,
65 depersonalisation, and a reduced sense of personal accomplishment (2). Recent
66 studies (1–4) have highlighted the growing impact of burnout in this profession as for
67 example, Biggs et al (3) revealed that 96% of UK physiotherapists experience
68 moderate to high levels of burnout, with perfectionism and moral injury being
69 significant predictors. This study underscores the urgent need for targeted
70 interventions to address burnout and its associated factors.
71 Although burnout is not a new concept, the COVID-19 pandemic has further
72 exacerbated these challenges, leading to increased workload and stress among
73 physiotherapists. Recent systematic reviews and meta-analyses have shown a
74 significant rise in burnout levels during the pandemic. For example, Clarke et al (5)
75 found that 40% of musculoskeletal allied health practitioners, including
76 physiotherapists, reported high levels of emotional exhaustion, with many attributing
77 this to the increased demands and stress brought on by the pandemic. Similarly,
78 Venturini et al (1) reported a global prevalence of burnout at 8%, highlighting the
79 widespread impact of this issue.
80 Burnout in physiotherapy has profound implications not only for the individuals
81 affected by it but also for the healthcare system as a whole. It can lead to decreased
82 job satisfaction, increased absenteeism, and higher turnover rates, which in turn affect
83 patient care and outcomes (2,6). Additionally, burnout can result in significant mental
84 health issues such as anxiety, depression, and substance abuse (7). These findings
85 align with the broader literature on healthcare professional burnout, which consistently
86 highlights the detrimental effects on both personal well-being and professional
87 performance (8).
88 Despite the substantial quantitative data on burnout prevalence and risk factors, there
89 is a gap in the literature regarding the qualitative experiences of physiotherapists,
90 particularly in the UK. Quantitative research has created a foundation by identifying
91 key factors such as perfectionism and moral injury (3,9) but did not delve into the
92 personal and emotional experiences of physiotherapists. Exploring the perceptions
93 and experiences of physiotherapists who have been impacted from burnout is crucial
94 as it allows for a deeper understanding of the nuances and complexities of this critical
95 issue in healthcare. It also provides insights into how burnout manifests in daily
96 practice, affects professional identity, and impacts personal well-being.
97 Given the high prevalence of burnout and its severe implications, this research is
98 timely and essential for advancing our understanding and support of physiotherapists
99 in the UK and beyond. Listening to physiotherapists' voices about their experiences
100 and perspectives is crucial for developing a comprehensive understanding of burnout
101 and the strategies they employ to manage it. By exploring these personal narratives,
102 we can gain insights into the real-world challenges faced by physiotherapists and the
103 coping mechanisms that may not be evident in quantitative studies alone.
104 Therefore, the aim of this study is to explore the lived experiences of UK
105 physiotherapists regarding burnout, perfectionism, and moral injury and to develop a
106 comprehensive understanding of the personal and professional impacts of these
107 issues and to inform the development of effective support systems and interventions.

## 108 Methods

109 **Design**
110 This study employed a generic qualitative approach to explore and understand the
111 experiences of UK physiotherapists regarding burnout, perfectionism, and moral injury.
112 A generic qualitative approach is well-suited for investigating phenomena or processes
113 without aligning with a specific qualitative methodology, allowing for a broad and
114 flexible exploration of clinical issues (10). This approach enabled researchers to
115 gather rich, detailed narratives that offer deeper insights into the complexities of
116 burnout and related factors. The methods utilised in this study involved semi-
117 structured interviews, which are highly effective for obtaining detailed insights into
118 participants' experiences. This format provided the necessary flexibility to delve into
119 physiotherapists’ perspectives while maintaining enough structure to ensure all key
120 topics are addressed
121 Ethical approval for the study was granted by the Glasgow Caledonian University
122 (HLS/PSWAHS/22/017), ensuring that all research activities were conducted in
123 accordance with ethical standards. The study was also guided by the Consolidated
124 Criteria for Reporting Qualitative Research (COREQ) checklist, which provided a
125 comprehensive framework for ensuring rigour and transparency in qualitative
126 research.

## 127 Participant Recruitment

128 To follow up on the “Physiotherapy Under Pressure” study conducted by Biggs et al (3)
129 participants were recruited using purposive sampling. This approach involved
130 selecting physiotherapists who had previously completed the survey and had
131 expressed interest in participating in a qualitative follow-up study. Inclusion criteria
132 included physiotherapists working in the UK, being over 21 years of age, working full-
133 time or part-time and having experienced burnout. Exclusion criteria applied to those
134 being retired or a student. All potential participants were contacted via email and
135 invited to participate in the study. Those who expressed interest were provided with
136 detailed information about the study and asked to consent to participate. Participants
137 were informed that consent was an ongoing process and that they could withdraw at
138 any time. This information was reiterated when the participant information sheet was
139 emailed during the recruitment process for the qualitative study. Written consent was
140 sought at this stage. During the interview, verbal consent was sought again at the
141 outset and was documented by the researcher.

## 142 Data Collection

143 Recruitment emails were sent out in October 2023 through to the end of January
144 2024, and data were collected through semi-structured interviews conducted online via
145 Zoom between November 2023 and March 2024. Each interview lasted between 45 to
146 60 minutes, allowing sufficient time to explore participants' experiences in depth. The
147 interview guide was developed based on a review of relevant literature and refined
148 through feedback from the research team and pilot interviews. A pilot study was
149 initially conducted with one participant, followed by a debrief session where the
150 qualitative researcher made notes for changes. Feedback from the pilot interview led
151 to minor adjustments in the wording and sequencing of questions to improve clarity
152 and flow. A second pilot study confirmed the final interview guide, as no further
153 changes were needed.
154 The interview guide consisted of open-ended questions designed to elicit detailed
155 narratives about participants' experiences with burnout, perfectionism, and moral
156 injury within their physiotherapy roles. Examples of questions included: "Can you
157 describe how burnout has impacted your professional performance?" and "What
158 support, if any, have you received from your workplace to manage these challenges?"
159 This approach ensured comprehensive coverage of relevant topics while allowing
160 participants to express their views and experiences freely. During the interviews, the
161 researcher took notes on body language, nonverbal responses, and the use of
162 metaphors to capture the full depth of participants' experiences. Interviews continued
163 until data saturation was achieved, meaning no new themes or insights were emerging
164 from the data (11). This approach ensured a comprehensive understanding of the
165 phenomena being studied.
166 To ensure the reliability and accuracy of the data, all interviews were recorded and
167 transcribed verbatim using Zoom. The transcripts were then meticulously reviewed
168 and edited by the qualitative researcher for accuracy. Data were stored securely in an
169 encrypted space on OneDrive, and all identifying information was removed to maintain
170 participant anonymity. Each participant was assigned a pseudonym (e.g., Participant
171 1, Participant 2) for the reporting process. Participants were informed of their right to
172 withdraw from the study at any time. They were encouraged to speak openly and were
173 given the option to take breaks or skip questions due to the sensitive nature of the
174 topic. After the interviews, the lead researcher took time to reflect and documented
175 detailed notes on the conversation and the significant points raised by the participants.
176 This additional context enriched the data, providing a deeper insight into the
177 participants' experiences.

## 178 Data Analysis

179 The data analysis followed a framework approach developed by Spencer et al (12),
180 comprising five intertwined stages. The analytic process began with the first author
181 familiarising herself with the data by reading and re-reading the transcripts to immerse
182 herself in the content. This was followed by the initial coding of the data. The
183 qualitative researcher gathered all similar data into clusters and created initial
184 categories. In total, 220 codes were created from the 12 interviews, and a coding
185 matrix was created in NVivo 14 for each of them. Identifying codes and clustering
186 them into categories was aided by mapping exercises, which helped visualise the
187 relationships between different codes. Descriptions of each category were created to
188 capture the essence of the data, and categories with similar meanings were then
189 further grouped under broader thematic titles. The labels and descriptions used for the
190 raw data were revisited and carefully examined to ensure they accurately reflected the
191 interview data.
192 During this iterative process, coded content was reviewed and, if necessary, moved or
193 created a combined different category. This thorough process generated nine final
194 categories, which were then further refined into four analytical overarching themes. To
195 enhance the rigour and transparency of the findings, a reflective diary was maintained
196 throughout the research process. This diary enabled the documentation of personal
197 biases, assumptions, and reflections, acknowledging how the researchers’
198 background and experiences might influence the interpretation. The final thematic
199 structure provided a comprehensive understanding of the experiences of UK
200 physiotherapists with burnout, perfectionism, and moral injury, contributing valuable
201 insights to the survey results (3) and potentially inform the development of
202 interventions and support strategies.

## 203 Results

204 **Participants Characteristics**
205 Twelve Physiotherapists took part in the study, including 8 females and 4 males, from
206 various specialties and organisational types. Data collected included age, gender,
207 area of physiotherapy, organisation type and length of service (Table 1). This diverse
208 sample provided a broad perspective on the experiences of physiotherapists across
209 different contexts.

## 210 Table 1: Participants characteristics

| **ID** | **Ag e** | **Gender** | **Area of Practice** | **Organisatio n Type** | **Banding Type** |
| --- | --- | --- | --- | --- | --- |
| Participant 1 | 32 | Female | Musculoskeletal | NHS Trust | Band 6 |
| Participant 2 | 45 | Male | Musculoskeletal | Private Practice | Band 7 |
| Participant 3 | 29 | Female | Musculoskeletal | Private Practice | Band 6 |
| Participant 4 | 34 | Female | Paediatrics | NHS Trust | Band 6 |
| Participant 5 | 50 | Male | First Contact Practitioner | GP surgery | Band 8b |
| Participant 6 | 38 | Female | Cardiorespiratory | NHS Trust | Band 6 |
| Participant 7 | 31 | Female | Orthopaedics | NHS Trust | Band 6 |
| Participant 8 | 46 | Male | Community | NHS Trust | Band 6 |
| Participant 9 | 38 | Female | First Contact Practitioner | Private Practice | Band 8a |
| Participant 10 | 42 | Male | Community | NHS Trust | Band 6 |
| Participant 11 | 35 | Female | Mental Health | NHS Trust | Band 7 |
| Participant 12 | 26 | Female | Rotation | NHS Trust | Band 5 |

211

212 Findings in this study are structured around four overarching themes: Physiotherapy
213 Under Pressure: Workload, Burnout, and Perfectionism, Interpersonal Dynamics and
214 Support Systems, Professional Fulfilment and Identity and Work-Life Balance and
215 Well-being.

## 216 Theme 1: Physiotherapy Under Pressure: Workload, Burnout, and Perfectionism

217 This theme explored the complex relationship between workload, burnout, and
218 perfectionism among UK physiotherapists. Participants’ narratives offered a detailed
219 insight into the pervasive issue of burnout, highlighting not just the immediate effects
220 of heavy workloads, but also the long-term implications for professional fulfilment and
221 personal well-being.

## 222 Burnout and Exhaustion

223 The high workloads faced by physiotherapists had severe impacts on their well-being,
224 leading to burnout and exhaustion. Participants frequently reported emotional and
225 physical exhaustion due to the relentless pace of their work. They often managed
226 numerous patients daily, with back-to-back appointments and minimal breaks in
227 between. This relentless schedule left little time for recovery, leading to physical
228 exhaustion. *"The back-to-back patient appointments with no time to breathe in*
229 *between have pushed me to my limits,"* shared one physiotherapist.
230 This lack of recovery time resulted in feeling constantly overwhelmed and struggling to
231 maintain their professional responsibilities. One participant highlighted the emotional
232 toll of the job, stating, *"There have been times when I felt so drained, both physically*
233 *and emotionally, that I could barely function."* This emotional exhaustion often led to
234 decreased motivation impacting their ability to provide quality patient care as the role
235 requires to continuously engage with patients, listen to their concerns and provide
236 emotional support.

## 237 The Aftermath of COVID-19

238 The COVID-19 pandemic exacerbated the challenges faced by physiotherapists,
239 significantly impacting their workloads, mental health, and the support they received
240 from management. This period brought an increase in both anxiety and workload, as
241 well as the need for significant organisational changes. Participants reported handling
242 more patients than ever, often under stressful conditions. *"The heightened anxiety*
243 *affected my mental health and work performance, making it challenging to maintain*
244 *professional responsibilities,"* noted one interviewee, indicating how the already high
245 demands of their job had been intensified. This increased pressure contributed
246 significantly to burnout, as physiotherapists found themselves stretched thin, both
247 physically and mentally.
248 The uncertainty and stress related to COVID-19 continued at the end of the pandemic.
249 Staffing challenges also became more pronounced during and after this era. Teams
250 were left short-staffed which further increased the burden on those who remained.
251 One participant shared, *"We were already dealing with high workloads, but with*
252 *colleagues off sick or quitting, it became nearly impossible to manage."*
253 The transition to remote work introduced a new set of challenges. Physiotherapists
254 had to adapt quickly to virtual consultations and new technologies, often without
255 adequate support from management. *"The lack of consistent support from*
256 *management during COVID-19 has made the situation even more stressful and*
257 *frustrating,"* noted one physiotherapist.
258 However, remote work remained after the pandemic and increased administrative
259 burdens. Many physiotherapists found themselves dealing with more paperwork and
260 bureaucratic processes than before which many felt they had not "signed up" to do. As
261 explained by one participant, *"I would spend hours on admin tasks, and then come*
262 *back to the clinic the next day only to find we were behind on patient care tasks. It felt*
263 *like we were constantly playing catch-up."* The need to juggle multiple roles left many
264 feeling they were unable to give their best to their patients, further contributing to
265 feelings of professional dissatisfaction and burnout.

## 266 Work Environment

267 The work environment emerged as a critical factor influencing the burnout and
268 resilience of physiotherapists. Inconsistent support from management was significantly
269 affecting physiotherapists' ability to manage their workloads and maintain their
270 motivation. Many participants expressed also feelings of isolation and frustration. One
271 physiotherapist said, *"The lack of consistent support from management makes it hard*
272 *to stay motivated."*
273 The absence of consistent management support was not only demoralising but also
274 led to a sense of helplessness exacerbating the already high levels of stress
275 associated with their role. Physiotherapists frequently found themselves navigating
276 complex and demanding situations without the necessary guidance or backing from
277 their managers. This lack of support contributed to a feeling of being undervalued and
278 increased the risk of burnout. Another participant noted, *"When management is not*
279 *there, you start to question why you are putting in all this effort."*
280 Bureaucratic challenges were frequently cited as significant sources of stress for
281 physiotherapists. The changes in protocols, coupled with the increasing administrative
282 workload, added layers of complexity to their daily routines. *"The bureaucracy and*
283 *constant changes in protocols are exhausting,"* shared one physiotherapist. These
284 hurdles not only increased the time spent on non-clinical tasks but also detracted from
285 the time available for patient care, which many physiotherapists found particularly
286 frustrating. They often felt disconnected from the practical realities of clinical work,
287 leading to inefficiencies and added stress. One physiotherapist explained, *"I spend*
288 *hours on paperwork that seems pointless, and it takes away from the time I should be*
289 *spending with patients."*
290 Participants called for better support systems as the current structures were often
291 seen as rigid and unsupportive, contributing to the high levels of stress and burnout.
292 Suggestions included streamlined processes for handling administrative tasks, better
293 communication from management, and the introduction of more flexible work
294 arrangements. One participant explained, *"The system needs an overhaul. We need*
295 *structures that support us, not ones that add to our stress."*
296 Finally, the impact of workload and burnout varied significantly across different career
297 stages, highlighting the unique challenges faced by physiotherapists at various points
298 in their professional journeys. Newly graduated physiotherapists often felt immense
299 pressure and lacked sufficient support. One participant shared a distressing
300 experience: *"I used to lock myself in the storage room to cry because I felt so much*
301 *pressure, and my seniors didn't have the time to help me—they only criticised."*
302 Similarly, senior physiotherapists also faced considerable challenges, often feeling
303 overwhelmed by their responsibilities. These included supervising students, managing
304 patients, provide training for others and handling bureaucratic tasks.

## 305 Perfectionism

306 Perfectionism was another critical factor that influenced the burnout and well-being of
307 physiotherapists. The high standards and expectations that physiotherapists set for
308 themselves often led to increased stress and emotional exhaustion. They explained
309 that felt inadequate and constantly strived to meet unattainable standards. *"I always*
310 *feel like I need to do everything perfectly, and it just wears me out,"* shared one
311 participant. This pursuit of perfection not only contributed to emotional exhaustion but
312 also affected their professional performance and personal life. One participant noted,
313 *"The constant need to be perfect in everything I do is exhausting. It feels like I'm never*
314 *good enough, no matter how hard I try. I just want to quit from life"*.
315 Some participants employed various strategies to cope with perfectionism, including
316 therapy, self-awareness practices, and developing self-compassion. However, other
317 challenges highlighting the difficulties in managing their high standards and
318 expectations. *"Sometimes, despite my efforts, I still find it hard to let go of my*
319 *standards,*" shared one physiotherapist.

## 320 Moral Injury

321 Physiotherapists often entered the profession with a strong sense of duty and
322 commitment to providing high-quality patient care. However, participants discussed
323 that systemic issues such as inadequate resources, high workloads, and bureaucratic
324 obstacles frequently prevent them from fulfilling their roles causing deep frustration.
325 One participant explained: *"It’s incredibly frustrating when I know what the best care*
326 *for my patient would be, but I skip it "* .
327 In addition, the ongoing struggle to reconcile professional values with the constraints
328 of the healthcare system often resulted in significant moral distress. Participants found
329 themselves in situations where they had to compromise on the quality of care due to
330 factors beyond their control, such as limited time, insufficient staffing, or restrictive
331 protocols. This led to feelings of guilt, shame, and dissatisfaction. For example, one
332 participant said, *"I feel like I'm constantly fighting against a system that doesn't care*
333 *about the patients or staff,"*.
334 The emotional and psychological consequences of moral injury were clear as
335 physiotherapists reported feelings of anger, betrayal, and disillusionment with the
336 healthcare system leading to burnout, and even a desire to leave the profession. One
337 participant explained *"I feel like I'm constantly battling the system. It’s draining and*
338 *makes me question why I chose this profession. And imagine that I was a mature*
339 *student and this was my dream job".*

## 340 Theme 2: Interpersonal Dynamics and Support Systems

341 This theme highlights the critical role that interpersonal relationships and support
342 systems play in the professional and personal lives of physiotherapists with burnout.
343 The narratives highlight the impact of team dynamics, support networks, interpersonal
344 challenges, and the emotional repercussions on the well-being and job satisfaction of
345 physiotherapists.

## 346 Team Dynamics

347 Team dynamics were crucial in shaping the work environment and overall experience
348 of participants. They explained that effective team support and interaction significantly
349 mitigates stress and burnout. Many participants emphasised the importance of having
350 a cohesive team where members understand and support each other. One participant
351 noted, *"Having a supportive team makes a huge difference. We look out for each other*
352 *and help manage the workload."*
353 However, not all participants experienced positive team dynamics. Some reported a
354 lack of understanding and support from their teams and managers, which exacerbated
355 their stress levels. *"The lack of consistent support from management makes it hard to*
356 *stay motivated,"* explained one interviewee. This inconsistency especially in
357 managerial support left them feeling isolated and overwhelmed, highlighting the need
358 for better leadership.
359 Effective communication within teams also emerged as a pivotal factor. Participants
360 who reported good communication within their teams felt more equipped to handle
361 their responsibilities and less isolated in their roles. *"Good communication within the*
362 *team helps us stay on the same page and manage the day efficiently,"* noted one
363 physiotherapist.

## 364 Support Networks

365 Support networks, both within and outside the workplace, were essential for the
366 emotional and professional well-being of physiotherapists. Participants highlighted the
367 benefits of having mentors or peers they could turn to for guidance. They explained
368 that their colleagues often serve as a primary source of support, providing both
369 emotional and professional assistance. *"Support from my colleagues has been*
370 *invaluable. They understand the pressures of the job and are always there to lend a*
371 *hand or offer advice,"* shared one participant.
372 In addition, emotional support systems extended beyond the workplace,
373 encompassing family and friends who provided a crucial safety net. *"Having support at*
374 *home helps me cope with the stresses of the job. My family and friends are my rock,"*
375 explained another interviewee. This external support was vital in helping
376 physiotherapists with burnout to manage the emotional toll of their work.

## 377 Interpersonal Challenges

378 Despite the benefits of strong support networks, many physiotherapists faced
379 significant interpersonal challenges. Being criticised and bullied by colleagues or
380 managers was a common experience that negatively impacted their job satisfaction
381 and mental health. *"I felt constantly criticised by my seniors, which made me doubt my*
382 *abilities and increased my stress levels,"* shared one participant. The impact of these
383 challenges was particularly pronounced for newly graduated physiotherapists who
384 often felt immense pressure and lack sufficient support.
385 Minimal team interaction and conflicts with colleagues further contributed to these
386 challenges. *"There were times when I felt completely isolated because my colleagues*
387 *were too busy to even talk to each other"* noted one physiotherapist. These
388 interpersonal conflicts not only hinder team cohesion but also increase individual
389 stress and frustration. Such negative interactions can create a toxic work environment,
390 leading to further isolation and burnout.

## 391 Emotional Impact

392 Physiotherapists frequently reported that the lack of support and negative interactions
393 led to feelings of isolation and emotional dysregulation. "*The constant criticism and*
394 *lack of support made me feel isolated and emotionally drained. I was wondering ‘what*
395 *am I doing here?”,"* explained one participant. This emotional toll affected their
396 professional performance but also spilled over into their personal lives, impacting their
397 overall well-being. Participants noted that the stress and emotional exhaustion from
398 work often strained their relationships with family and friends. *"By the end of the day,*
399 *I’m so exhausted that I can barely spend time with my family"* shared another
400 physiotherapist.

## 401 Theme 3: Professional Fulfilment and Identity

402 This theme highlighted the multifaceted nature of professional fulfilment and identity
403 among UK physiotherapists. The narratives from the interviews revealed the
404 complexities of finding professional satisfaction in a demanding and often undervalued
405 profession.

## 406 Recognition and Appreciation

407 Recognition and appreciation from patients and colleagues were crucial for
408 professional fulfilment among participants. The acknowledgment of their efforts
409 provides a sense of validation and boosts morale. One participant expressed, *"It's*
410 *been appreciated either you know by patients or sometimes by colleagues that you've*
411 *been helpful."* This appreciation not only reinforced their professional efforts but also
412 fostered a sense of belonging within the professional community.
413 Appreciation in work also contributed to job satisfaction. Physiotherapists who felt
414 valued in their roles reported higher levels of motivation and commitment. Being
415 recognised for their contributions was not just a morale booster but a critical aspect of
416 their professional identity. One interviewee shared, *"Recognition by patients and*
417 *colleagues makes a big difference. It feels like all the hard work is acknowledged and*
418 *appreciated. Then I know I am at the right place".*

## 419 Sense of Accomplishment

420 Physiotherapists derived significant satisfaction from their ability to make a difference
421 in their patients' lives. This sense of accomplishment was closely tied to their
422 confidence in their professional roles. One participant shared, *"Feeling useful and*
423 *productive in your work helps maintain motivation and purpose…That I am in the right*
424 *place and I can do this and I can do that*.." reflecting the intrinsic motivation and self-
425 assurance that came from performing well in the job. Participants often spoke about
426 the joy and fulfilment they experienced when their efforts led to positive patient
427 outcomes. *"When a patient tells me that they feel better because of my treatment, it*
428 *makes all the hard work worth it,"* said one physiotherapist.

## 429 Career Development and Growth

430 Many physiotherapists expressed that while continuous professional development
431 (CPD) is essential for maintaining the requirements of their professional body and
432 enhancing their skills, the demands of their workload left them feeling overwhelmed
433 and exhausted. One participant highlighted, *"Continuous learning is crucial for my*
434 *growth and to feel confident in my role, but the expectations and time commitment can*
435 *be daunting."* This sentiment was echoed by others who felt that the time and effort
436 required for CPD, on top of their already demanding workloads, contributed to their
437 burnout.
438 Moreover, the expectations set by professional bodies for CPD often felt unrealistic
439 and added to the sense of pressure. *"The professional bodies have set such high*
440 *standards for CPD, and it feels like there’s no flexibility. It just adds another layer of*
441 *stress, and they are oblivious to what is happening to us and how demanding is our*
442 *profession"* remarked another participant. The lack of structured and supportive CPD
443 opportunities within their organisations also emerged as a concern. Many
444 physiotherapists reported that they had to pursue CPD on their own time, which often
445 encroached on their personal time and work-life balance. *"We’re expected to do all*
446 *this CPD, but there’s no time allocated for it during work hours. It’s like we’re expected*
447 *to do it in our own time, which just adds to the burnout,"* noted a participant.
448 Working outside of their roles to create opportunities was a strategy employed by
449 some physiotherapists to overcome the lack of formal career progression pathways.
450 They took on additional responsibilities or pursued further education to broaden their
451 career prospects. *"I had to take extra courses on my own time to stay ahead," shared*
452 *one participant*". However, the absence of structured career progression opportunities
453 often led to frustration and a feeling of being undervalued. Another participant
454 articulated, *"The lack of career progression opportunities makes me feel stagnant. I*
455 *need new challenges to stay motivated."*

## 456 Career Stagnation and Change

457 Career stagnation was a significant issue, with many physiotherapists feeling stuck in
458 the same role for extended periods. This lack of movement not only hindered their
459 professional growth but also contributed to job dissatisfaction. Participants discussed
460 the desire for career change as a natural response to this stagnation. Some
461 contemplated shifting careers entirely to burnout and others to escape the monotony
462 and seek new challenges. This desire was driven by the need for professional
463 fulfilment and the aspiration to find a role that offered better growth prospects. One
464 participant shared. *"I've been doing the same thing for years now, and it feels like I'm*
465 *just spinning my wheels. I'm seriously thinking about switching careers because I need*
466 *something new and challenging to keep me motivated and balanced."*
467 Some physiotherapists considered shifting to different roles within healthcare or
468 entirely different fields. *"I’ve thought about leaving physiotherapy altogether because I*
469 *don’t see any future growth here,"* shared one participant. This desire reflected the
470 need for a more supportive workplace that offers opportunities for career advancement
471 and work-life balance. "I’ve been doing the same thing for years, and it feels like
472 there’s no way out," noted one interviewee.

## 473 Theme 4: Work-Life Balance and Well-being

474 This theme captured the multifaceted aspects of work-life balance, coping
475 mechanisms and the importance of well-being among physiotherapists. The insights
476 provided by the participants highlight the critical need for systemic changes to support
477 physiotherapists in achieving a healthier balance between their professional and
478 personal lives.

479

## 480 Work-Life Integration and Flexibility

481 Participants frequently highlighted the challenges of maintaining a healthy work-life
482 balance. The demanding nature of their job often led to poor work-life balance, with
483 many struggling to separate their professional responsibilities from personal time. One
484 participant remarked, *"It's hard to switch off after a long day at work. I often find myself*
485 *thinking about patients and pending tasks even when I'm home."* The struggle with
486 maintaining work-life balance was evident as many physiotherapists found their
487 personal lives impacted by their professional commitments. *"I used to have hobbies*
488 *and interests outside work, but now I hardly find the time or energy for them,"* shared
489 by another participant.
490 Flexibility in scheduling and the ability to set boundaries emerged as crucial factors for
491 achieving better work-life integration. *"Being able to adjust my schedule and take*
492 *breaks when needed has been a game-changer for my mental health,"* noted one
493 physiotherapist. This flexibility (where possible) allowed them to manage their
494 workload more effectively and allocate time for personal activities, thereby reducing
495 stress.
496 However, this was not always supported by the organisational structures in place.
497 Many participants expressed the need for better support systems and resources to
498 help manage their workloads. "We need more support from the management to
499 ensure that breaks are respected, even if this is for lunch, a walk or a counselling
500 session" emphasised a participant, highlighting a gap in current practices.

## 501 Personal Well-being and Coping Mechanisms

502 The toll on personal well-being was a recurrent issue, with many physiotherapists
503 reporting mental health struggles, physical health symptoms, and sleep disturbances.
504 *"The constant stress and pressure have led to frequent headaches and insomnia,"*
505 shared one participant, highlighting the physical manifestations of work-related stress.
506 Emotional resilience was identified as a key factor in managing these challenges.
507 Developing self-compassion and seeking professional help, such as therapy, were
508 common coping strategies. *"Therapy has been instrumental in helping me deal with*
509 *the emotional burden of my job,"* noted one physiotherapist.
510 Exercise and self-awareness practices were also frequently mentioned as effective
511 coping mechanisms. *"Regular exercise has been my go-to stress buster. It helps me*
512 *clear my mind and stay physically fit,"* shared a participant. Another added, *"Practicing*
513 *mindfulness and staying aware of my emotional state has been crucial in managing*
514 *my stress levels."* Despite these efforts, many physiotherapists still struggled to find a
515 balance, indicating the need for more systemic support. *"No matter how many coping*
516 *strategies I employ, the workload and pressure sometimes feel overwhelming,"*
517 confessed one participant, highlighting the limitations of individual efforts in the
518 absence of broader organisational changes.

## 519 Management Respecting Work-Life Balance

520 The role of management in respecting and supporting work-life balance was
521 emphasised by many participants. The need for better mental health support and
522 resilience-building initiatives from management was a recurring theme. *"We need*
523 *management to acknowledge the stress we are under and provide resources and the*
524 *time to support our mental health"* stated one physiotherapist.
525 Support from family and friends played a critical role in helping physiotherapists cope
526 with their demanding jobs. *"My family has been my rock. Their support keeps me*
527 *going,"* shared one participant, illustrating the importance of a strong personal support
528 network. However, there was a clear call for more structured support from the
529 workplace to complement these personal networks. Participants also expressed a
530 need for management change and training to foster a more supportive work
531 environment. This included providing more resources, improving communication, and
532 creating a culture that values and respects work-life balance. *"Management needs to*
533 *listen to our concerns, recognise burnout and take concrete steps to reduce our*
534 *workload and stress,*" emphasised by one physiotherapist.

## 535 Discussion

536 This study provides an in-depth exploration of the factors contributing to burnout
537 among UK physiotherapists. The narratives revealed the intricate interplay between
538 workload, burnout, perfectionism, and the professional identity of physiotherapists,
539 offering critical insights into the systemic issues affecting their well-being and coping
540 strategies as well.
541 Burnout and workload are significant, interconnected issues. The high demands
542 placed on physiotherapists often lead to severe emotional and physical exhaustion.
543 This aligns with the broader literature, indicating that excessive work demands are a
544 primary driver of burnout in healthcare professionals (9,13,14). The relentless pace,
545 lack of adequate breaks, and the need to juggle multiple roles detract from their
546 capacity to provide high-quality patient care and maintain personal well-being. This is
547 not merely an issue of individual resilience; it points to systemic flaws within
548 healthcare organisations that fail to support their staff adequately. These include
549 inadequate support structures, inefficient work processes, and a lack of recognition of
550 the critical need for rest and recovery among healthcare professionals (2). By
551 recognising and addressing these systemic issues, healthcare organisations can
552 create a more sustainable and supportive work environment that promotes both the
553 well-being of physiotherapists and the quality of care provided to patients.
554 The COVID-19 pandemic has exacerbated these challenges, introducing new
555 stressors such as increased patient loads, the transition to remote work, and additional
556 administrative burdens (15). Greenberg et al (16) elaborates on how healthcare
557 professionals faced unprecedented pressures during the pandemic, leading to moral
558 injury and mental health problems due to the impossible decisions they had to make,
559 such as allocating scarce resources and balancing their own health needs with those
560 of their patients. The authors also emphasise the importance of proactive mental
561 health support and the implementation of routine support processes, such as peer
562 support programs, to mitigate the negative impacts of moral injury and mental health
563 issues. However, the issues faced by physiotherapists did not cease with the
564 pandemic; they continued as demands increased, staff numbers reduced, and funding
565 decreased. The lack of consistent management support during and after this period
566 further underscores the systemic issues at play. Healthcare organisations must invest
567 in long-term strategies to support their workforce to withstand such crises and beyond.
568 Perfectionism is another critical factor contributing to burnout (17).The constant
569 striving for perfection, while initially promoting high performance and meticulous care,
570 eventually leads to significant stress and emotional fatigue. Our findings highlight that
571 physiotherapists' high standards and expectations often lead to emotional exhaustion
572 and feelings of inadequacy. Koutra et al (18) explained that perfectionism is strongly
573 associated with increased psychological distress. This is compounded by the lack of
574 self-compassion, which can mediate the relationship between perfectionism and
575 psychological distress. Self-compassion allows individuals to treat themselves with the
576 same kindness and understanding they would offer to a friend in distress, thus
577 mitigating the negative effects of perfectionism. Addressing perfectionism through
578 interventions such as therapy and self-compassion practices can help mitigate its
579 negative impact (18). However, these individual-level interventions must be
580 complemented by systemic changes that reduce the pressure to meet unrealistic
581 standards. Healthcare organisations must acknowledge the detrimental effects of
582 perfectionism and take proactive steps to address it. This could include providing
583 training, offering resources and time for therapy and mental health support, and
584 fostering a work environment that values continuous improvement over flawless
585 performance.
586 Similarly moral injury emerged as a significant issue, stemming from the conflict
587 between professional values and the constraints of the healthcare system. This
588 concept is well-documented in literature, where healthcare professionals face
589 situations that force them to act against their moral or ethical beliefs due to systemic
590 constraints (19). Physiotherapists reported feelings of guilt, shame, and
591 dissatisfaction, which are common symptoms of moral injury. These emotions
592 contribute to burnout and increase the likelihood of professionals considering leaving
593 the profession (20). While personal coping strategies such as therapy are beneficial,
594 healthcare organisations need to establish robust support systems that can help
595 physiotherapists navigate the ethical dilemmas they face daily.
596 Team dynamics and support systems play a crucial role in mitigating stress and
597 burnout (21). Effective team dynamics and support from colleagues, family, and
598 friends are essential for emotional and professional well-being. Our findings
599 highlighted that physiotherapists face significant interpersonal challenges, such as
600 criticism and bullying, which negatively impact their job satisfaction and mental health.
601 The importance of supportive relationships in the workplace is well-supported by
602 research, which shows that strong professional and personal support networks can
603 buffer against the negative effects of job stress (22). Social support is crucial for
604 maintaining mental health and well-being, acting as a buffer against the stresses of the
605 job (22). This is particularly important in high-stress environments like healthcare,
606 where the demands can be overwhelming. Healthcare organisations need to create
607 work environments that encourage positive team dynamics and provides adequate
608 support to staff of all grades. Implementing strategies to promote teamwork, open
609 communication, and mutual respect can enhance job satisfaction and reduce burnout
610 among physiotherapists.
611 The demanding nature of physiotherapy frequently results in poor work-life balance,
612 with many professionals struggling to delineate their professional responsibilities from
613 personal time (1,3,23). This lack of balance is a significant contributor to burnout and
614 turnover intentions. Current organisational structures often fail to support necessary
615 flexibility in scheduling and the establishment of boundaries, exacerbating this issue.
616 This is consistent with findings that a lack of work-life balance contributes significantly
617 to burnout and turnover intentions among healthcare professionals (2,24). Fostering a
618 culture that values and respects work-life boundaries is crucial. This could involve
619 training for management on the importance of work-life balance, ensuring that regular
620 breaks and time off are respected, and providing resources for mental health support.
621 Encouraging open dialogue about the challenges of maintaining work-life balance can
622 also help in identifying specific areas where improvements are needed. This not only
623 enhances their well-being and job satisfaction but also improves patient care
624 outcomes, leading to a more resilient and effective healthcare system.

## 625 Conclusion

626 This study provides a comprehensive understanding of the complex relationship
627 between workload, burnout, and perfectionism among UK physiotherapists.
628 Addressing these issues requires systemic changes to support physiotherapists in
629 managing their workloads and maintaining their well-being. By implementing effective
630 policies, training, promoting professional development, and fostering supportive work
631 environments, healthcare organisations can reduce burnout and improve the
632 professional fulfilment and job satisfaction of physiotherapists, ultimately leading to
633 better patient care and outcomes.

## 634 References

635 1.	Venturini E, Ugolini A, Bianchi L, Di Bari M, Paci M. Prevalence of burnout
636 among physiotherapists: a systematic review and meta-analysis. Physiother
637 (United Kingdom) [Internet]. 2024;124:164–79. Available from:
638 https://doi.org/10.1016/j.physio.2024.01.007
639 2.	De Hert S. Burnout in healthcare workers: Prevalence, impact and preventative
640 strategies. Local Reg Anesth. 2020;13:171–83.
641 3.	Biggs D, Blackburn L, Black C, Shanmugam S. Physiotherapy Under Pressure:
642 A Cross-Sectional Study on The Interplay Between Perfectionism, Moral Injury,
643 and Burnout. medRxiv. 2024;1–29.
644 4.	Nozedar L, O’Shea S. What is the prevalence of burnout amongst first contact
645 physiotherapists working within primary care? Musculoskeletal Care.
646 2023;21(3):776–85.
647 5.	Clarke M, Frecklington M, Stewart S. Prevalence and Severity of Burnout Risk
648 Among Musculoskeletal Allied Health Practitioners: A Systematic Literature
649 Review and Meta-analysis. Occup Heal Sci. 2024;
650 6.	Kelly LA, Gee PM, Butler. Impact of nurse burnout on organizational and
651 position turnover. Nurs Outlook. 2021;69(1):96–102.
652 7.	Duarte I, Teixeira A, Castro L, Marina S, Ribeiro C, Jácome C, et al. Burnout
653 among Portuguese healthcare workers during the COVID-19 pandemic. BMC
654 Public Health. 2020;20(1):1–10.
655 8.	West CP, Dyrbye LN, Shanafelt TD. Physician burnout: contributors,
656 consequences and solutions. J Intern Med [Internet]. 2018 Jun 1;283(6):516–29.
657 Available from: https://doi.org/10.1111/joim.12752
658 9.	Burri SD, Smyrk KM, Melegy MS, Kessler MM, Hussein NI, Tuttle BD, et al. Risk
659 factors associated with physical therapist burnout: a systematic review.
660 Physiother (United Kingdom) [Internet]. 2022;116:9–24. Available from:
661 https://doi.org/10.1016/j.physio.2022.01.005
662 10.	Caelli K, Ray L, Mill J. “Clear as mud”: Toward generic qualitative research. Int J

663	Qual Methods 2 (2) Artic 1. 2003;2(2):816–9.

664	11.	Bryman A. Social Research Methods. 5th ed. Oxford: Oxford University Press;

665	2016. 1–732 p.

666 12.	Spencer L, Ritchie J, Lewis J, Dillon L. Quality in Qualitative Evaluation: A
667 framework for assessing research evidence. Soc Res (New York) [Internet].
668 2003;1–123. Available from: <http://www.civilservice.gov.uk/wp->
669 content/uploads/2011/09/Quality-in-qualitative-evaulation_tcm6-38739.pdf
670 13.	Van den Broeck A, Vander Elst T, Baillien E, Sercu M, Schouteden M, De Witte
671 H, et al. Job demands, job resources, burnout, work engagement, and their
672 relationships: an analysis across sectors. J Occup Environ Med.
673 2017;59(4):369–76.
674 14.	Norful AA, Rosenfeld A, Schroeder K, Travers JL, Aliyu S. Primary drivers and
675 psychological manifestations of stress in frontline healthcare workforce during
676 the initial COVID-19 outbreak in the United States. Gen Hosp Psychiatry.
677 2021;69:20–6.
678 15.	Costin A, Roman AF, Balica R-S. Remote work burnout, professional job stress,
679 and employee emotional exhaustion during the COVID-19 pandemic. Front
680 Psychol. 2023;14:1193854.
681 16.	Greenberg N, Docherty M, Gnanapragasam S, Wessely S. Managing mental
682 health challenges faced by healthcare workers during covid-19 pandemic. bmj.
683 2020;368.
684 17.	Rogan S, Verhavert Y, Zinzen E, Rey F, Scherer A, Luijckx E. Risk factor and
685 symptoms of burnout in physiotherapists in the canton of Bern. Arch Physiother
686 [Internet]. 2019;9(1):19. Available from: https://doi.org/10.1186/s40945-019-
687 0072-5
688 18.	Koutra K, Mouatsou C, Psoma S. The Influence of Positive and Negative
689 Aspects of Perfectionism on Psychological Distress in Emerging Adulthood:
690 Exploring the Mediating Role of Self-Compassion. Behav Sci (Basel).
691 2023;13(11).
692 19.	Webb EL, Ireland JL, Lewis M, Morris D. Potential Sources of Moral Injury for
693 Healthcare Workers in Forensic and Psychiatric Settings: A Systematic Review
694 and Meta-ethnography. Trauma, Violence, Abus. 2024;25(2):918–34.
695 20.	Dean W, Talbot S, Dean A. Reframing Clinician Distress: Moral Injury Not
696 Burnout. Fed Pract [Internet]. 2019;36(10):447. Available from:
697 http://www.ncbi.nlm.nih.gov/pubmed/31892772%0A[http://www.pubmedcentral.ni](http://www.pubmedcentral.ni/)
698 h.gov/articlerender.fcgi?artid=PMC6913609
699 21.	Jun J, Costa DK. Is It Me or You? A Team Approach to Mitigate Burnout in
700 Critical Care. Crit Care Nurs Clin North Am [Internet]. 2020 Sep;32(3):395–406.
701 Available from: https://linkinghub.elsevier.com/retrieve/pii/S0899588520300423
702 22.	Drageset J. Social Support. In: Health Promotion in Health Care – Vital Theories
703 and Research [Internet]. Cham: Springer International Publishing; 2021. p. 137–
704 44. Available from: <http://connect.springerpub.com/lookup/doi/10.1891/0739->
705 6686.6.1.85
706 23.	Patel RM, Bartholomew J. Impact of job resources and job demands on burnout
707 among physical therapy providers. Int J Environ Res Public Health. 2021;18(23).
708 24.	National Academies of Sciences, Engineering and Medicine. Taking Action

| 709 | Against Clinician Burnout [Internet]. Washington, D.C.: National Academies |
| --- | --- |
| 710 | Press; 2019. 334 p. Available from: https:/[/www.n](http://www.nap.edu/catalog/25521)a[p.edu/catalog/25521](http://www.nap.edu/catalog/25521) |
| 711 |  |

COREQ Cheklist

[Click here to access/download](https://www2.cloud.editorialmanager.com/pone/download.aspx?id=38069250&guid=21140f4f-3157-401d-8680-e967ab5d73f6&scheme=1)

[Supporting Information COREQ Checklist.docx](https://www2.cloud.editorialmanager.com/pone/download.aspx?id=38069250&guid=21140f4f-3157-401d-8680-e967ab5d73f6&scheme=1)

Data

# [Click here to access/download](https://www2.cloud.editorialmanager.com/pone/download.aspx?id=38069251&guid=8f9b1902-9a02-40b8-b411-1f556893e3d0&scheme=1)

[Supporting Information - Compressed/ZIP File Archive Burnout Data.docx](https://www2.cloud.editorialmanager.com/pone/download.aspx?id=38069251&guid=8f9b1902-9a02-40b8-b411-1f556893e3d0&scheme=1)
