# Supplementary material for: Exploring burnout, perfectionism, and moral injury among UK physiotherapists: A qualitative study on professional fulfilment and well-being
Source: PLoS One. 2025 Feb 13;20(2):e0313730. doi: 10.1371/journal.pone.0313730 (PMC11825001; doi:10.1371/journal.pone.0313730)
Supplement: Attachment — Submitted filename: Response to Reviewers.docx [file pone.0313730.s005.docx]

# Reviewer 1 Comments and Responses

| **Comment** | **Response** | **Revised Manuscript** |
| --- | --- | --- |
| Listing the reasons behind burnout due to pandemic or COVID-19, will further enhance the relevance of the point. Line 73-75 | Thank you for the comment. This has been now amended. | Line 74-77 |
| Including policy recommendations to government for developing strategies/training programs to reduce burn out among UK physiotherapists, will help in applying the findings of the study. Line 107 | Thank you for the comment. While qualitative research cannot claim to directly inform policy, it plays a crucial role in enriching our understanding of the lived experiences of those affected, thereby offering valuable insights into the complexities of burnout among physiotherapists. These findings can provide a foundation for evidence-based discussions and potentially support the development or refinement of government strategies and policies. workplace reforms. | Line 107-114 |
| Flowchart of coded clusters into different 9 categories and 4 broader themes if depicted in flowchart pattern will be more visually clear Line 192-194 | Thank you for this suggestion. The coding process was conducted using NVivo software, where codes, categories, and themes were organized using a tree-like folder structure inherent to the software. Unfortunately, the visual representation generated by a print-screen in NVivo is of low quality and does not meet publication standards. For this reason, we have decided not to include this in the manuscript. However, to enhance transparency and provide clarity, we have included a supplementary document the final codes categories and themes creation. | Supplementary file. |
| Brief description of specialties diversity will enhance the varied perspectives. Line 205-206 | A description of the specialties represented by participants is already visible in table 1. | Table 1 |
| Listing of the challenges faced like shortage of staff or resources etc. during COVID resulted into burnout will further justify the point. Line 240-241 | The challenges faced during COVID-19, such as staff shortages and resource limitations, to support the point. However, further detailed analysis of these challenges is not included in the results section, as this is beyond the scope of the current study. This study focuses on the lived experiences of physiotherapists rather than an extensive discussion of COVID-19, which has already been widely addressed in existing research. | Line 250-251 |
| Can include words like “Formal” and “Informal support system” Line 369-373 | Thank you for the comment. This has been now amended. | Line 378-380 |
| “Constructive criticism” was there or “bullying” needs to be justified. Line 379-381 | The distinction between our understanding of constructive criticism and bullying via the voice of our participants has been clarified. | Line 393-395 |
| Relate how emotional dysregulation results into professional isolation Line 392-393 | Thank you for the comment. This has been now amended. | Line 410-412 |
| Examples of expectations set by professional bodies for CPD will be helpful for understanding the point. Line 438-439 | Thank you for the comment. This has been now amended. | Line 458-460 |
| Listing of career change options as escape to avoid stagnation will further enhance the efficacy of the point. Line 459-460 | Thank you for the comment. This has been now amended. | Line 481-483 |
| Quantifying extra work hours to substantiate the demanding nature of the job Line 482-484 | A comment has been added. | Line 507-508 |
| Listing of unrealistic standards and interventions to reduce them can be further more specified. Line 579-581 | A comment has been added. | Line 605-607 |

**Reviewer 2 Comments and Responses**

| **Comment** | **Response** | **Revised Manuscript** |
| --- | --- | --- |
| sample size is very less also sampling strategies has not been mentioned clearly | Thank you for this observation. Qualitative research prioritises depth and richness of data over large sample sizes. The sample size of 12 participants was deemed sufficient as data saturation was achieved, with no new themes emerging during analysis. This approach aligns with established qualitative research practices (Guest et al., 2006). Additionally, the sampling strategy has been clarified in the manuscript. Participants were purposively selected based on their completion of a prior survey and their willingness to participate in follow-up qualitative interviews, ensuring relevance to the research objectives. | Line 213-217 |
| on the basis of exploratory study the sample size is way to less as far as the statement is concern it can be validate on the basis of very less participant | As this is an exploratory qualitative study, the focus is on depth and richness of data rather than generalisability. The sample size of 12 participants is consistent with qualitative research standards, particularly when data saturation is achieved. Saturation ensures that no new themes emerge from the data, validating the sufficiency of the sample size for the study’s objectives. This approach is well-supported in the qualitative research literature. | No changes were made |
| As group has very different categories as 26 years old burnout may vary with 50 years old physiotherapist | Thank you for these insightful comments. The diversity in participants’ ages was considered during the analysis, and while some age-related variations were observed, the themes were consistent across the sample. These variations were acknowledged as contextually rich elements that enhance the understanding of burnout and moral injury within the profession. However, this study does not aim to compare specific age groups but to explore the overarching experiences of physiotherapists. | No changes were made |
| also the age may reflect to the moral injury with the age group of 26 and 50 due to aging and age related changes |  |  |
| author should also mention about the MSDs and evaluation aspects of the same | Thank you for this suggestion. The focus of this study is on the psychological impacts of burnout, perfectionism, and moral injury among physiotherapists. While musculoskeletal disorders may be relevant to the physical well-being of physiotherapists, this falls outside the scope of the current research objectives. The mention of physical exhaustion is contextualised here as a contributing factor to burnout rather than as an evaluation of MSDs. | No changes were made |
| A reference is needed to support the statement regarding the importance of Continuous Professional Development (CPD) and its impact on physiotherapists. | A reference has been added to support the statement regarding the essential nature of CPD and its challenges for physiotherapists. | Line 451 |
| Citation has not been done | Direct quotes from participants are used in qualitative research to highlight lived experiences and provide authenticity to the findings. As such, participant quotes are not typically cited with external references, as they represent primary data collected in this study. | No changes were made |
